# Contesting and Integrating Multiple Ways of Knowing in Collaborative Natural Resource Governance

**DOI:** 10.1007/s00267-026-02558-2

**Published:** 2026-07-22

**Authors:** Molly C. Levy, Vicken Hillis, Hailey Wilmer, Lauren M. Porensky, Dave Pellatz, Rebecca Som Castellano

**Affiliations:** 1 Human-Environment Systems, Boise State University, Boise, ID USA; 2 USDA-Agricultural Research Service Range Sheep Production Efficiency Research, US Sheep Experiment Station, Dubois, ID USA; 3 USDA-Agricultural Research Service, Rangeland Resources and Systems Research Unit, Fort Collins, CO USA; 4 Thunder Basin Grassland Prairie Ecosystem Association, Bill, WY USA; 5 Sociology, Boise State University, Boise, ID USA

**Keywords:** Collaborative governance, Ways of knowing, Natural resource management, Knowledge integration, Public lands, Stakeholder engagement

## Abstract

Collaborative governance is increasingly used to address complex natural resource issues, yet integrating diverse ways of knowing remains a persistent challenge. This study investigates how stakeholders engage with scientific, experiential, and administrative ways of knowing (WoKs) in a collaborative effort to manage public rangelands in the Thunder Basin ecoregion of Wyoming, USA. Drawing on qualitative data from interviews, a focus group, and participant observation, we examine how participants articulate and engage with different WoKs to inform land management goals, shape perceptions of knowledge legitimacy, and influence attitudes toward collaboration. Findings suggest that knowledge is strategically interpreted and relationally constructed through participants’ institutional roles, values, and social positions. While collaboration created opportunities for mutual learning, it also surfaced tensions around credibility, authority, and representation. Participants who were open to epistemic humility and treated knowledge as a tool for governance were better positioned to foster integration. These findings contribute to our understanding of collaborative governance by illustrating how epistemic dynamics shape interaction, learning, and decision-making within a multi-stakeholder process. More broadly, this case contributes to a growing recognition that meaningful collaboration requires moving beyond stakeholder diversity to critical engagement with how knowledge is produced, negotiated, and used in decision-making.

## Introduction

Management of public lands in the American West is complex and often contentious due to competing interests, ecological concerns, and historical legacies (Keiter and McKinney, 2019). A range of factors, including competing land uses, legal and regulatory complexity, environmental and economic pressures, and sources of knowledge, have the potential to contribute to overlapping and sometimes contradictory management objectives. Consequently, decision-making processes related to environmental governance frequently become sites of social, legal, or even physical conflict where divergent interests, values, and ways of knowing collide (Reid et al., 2006; Armitage et al., 2011; Tengö et al., 2014).

Collaborative governance is often posited as an effective way to approach management conflicts due to its potential to integrate diverse ways of knowing into decision-making processes. Recognizing and incorporating multiple forms of knowledge, such as scientific, professional, and local, is increasingly seen as essential for informed and adaptive governance (Berkes, 2009; Cash et al., 2003). However, while collaborative governance is meant to be an inclusive and deliberative process, frictions may result between different epistemological traditions. For instance, scientific expertise, bureaucratic knowledge, and place-based, experiential understandings may come into conflict, revealing challenges for effective knowledge integration (Hill et al., 2020; Wyborn et al., 2019). These tensions are exacerbated by power imbalances, competing values, and institutional constraints that shape the extent to which diverse knowledge is acknowledged and utilized (Hill et al., 2020; Wyborn et al., 2019).

Much of the existing literature on collaborative governance examines the conditions that contribute to either the success or failure of collaboration, focusing on structural and procedural factors such as stakeholder participation, institutional support, and trust-building (Ansell and Emerson et al., 2012; Gash, 2008; Koontz et al., 2015). However, less attention has been paid to the sociocultural dimensions that shape how collaboration unfolds in practice. Sociocultural, political, and institutional contexts influence how stakeholders engage with one another, interpret information, and navigate conflict. In particular, differences in how stakeholders produce, access, and legitimize knowledge can shape governance processes and outcomes (Brugnach, 2017; Nightingale, 2016). Exploring these dynamics provides a useful lens for examining how collaborative governance processes unfold in specific contexts, including the extent to which they support meaningful and equitable participation across diverse ways of knowing.

This study examines the role of diverse ways of knowing in collaborative natural resource governance through a case study of a multi-stakeholder working group convened to inform public rangeland management and planning (on a US Forest Service National Grassland in Wyoming, USA) in the context of environmental disturbance. Drawing on participant observation, a focus group, and semi-structured interviews, we analyze how participants articulated and engaged with a range of knowledge sources, including scientific expertise, lived experience, professional practice, and place-based knowledge. We examine how these ways of knowing were associated with different management priorities and how participants perceived, engaged with, and responded to them during the collaborative process.

By analyzing participants’ reflections and interactions, this study explores how diverse ways of knowing can shape collaborative governance, particularly in terms of how knowledge is perceived, valued, and integrated in defining goals and evaluating success. Through this investigation, we contribute to ongoing discussions about how diverse forms of knowledge influence collaborative decision-making in natural resource governance (Armitage et al., 2011; Berkes, 2009; Reed et al., 2018). This study advances understanding of the dynamics of collaborative governance through an in-depth, small-n case study, offering insight into how ways of knowing are valued, negotiated, and integrated within a multi-stakeholder collaboration.

## Background

### Navigating Knowledge Diversity in Collaborative Governance

Collaborative governance has become a widely adopted approach for addressing complex environmental challenges, particularly in contexts where diverse stakeholders must navigate competing knowledge, interests, and relationships regarding shared resources (van Tol Smit et al., 2015), though how it functions in practice varies across contexts. In the American West, collaboration is especially common in public lands management, where a history of conflict over land use, identity, and access has created a need for more inclusive and durable decision-making processes such as those offered by place-based collaboratives (e.g., Malpai Borderlands Group [Allen 2006], Quivira Coalition [White 2003], Collaborative Forest Landscape Restoration Program [Urgenson et al., 2017]). Defined by its emphasis on participatory and non-hierarchical decision-making, collaborative governance aims to foster legitimacy, trust, and social learning among governmental, non-governmental, and community actors (Ansell and Emerson et al., 2012; Gash, 2008; van Tol Smit et al., 2015). The growing adoption of collaborative governance in recent decades has been driven in part by declining trust in top-down management approaches, the increasing complexity of socio-ecological problems, and policy mandates that encourage stakeholder participation and decentralized decision-making (Wondolleck and Yaffee, 2000; Emerson et al., 2012; Koontz et al., 2015).

Collaborative governance is not a single model but can take multiple forms in relation to institutional arrangements, histories of conflict, and the particular actors involved. It can range from loosely structured partnerships to co-management institutions with formal decision-making authority. Regardless of its structure, these processes aim to produce context-sensitive solutions that enhance compliance, improve ecological resilience, and mitigate long-standing conflicts (Berkes, 2009; Folke et al., 2005). However, the success of collaborative governance is contingent upon more than assembling diverse stakeholders; it also requires negotiating underlying differences in how those stakeholders understand and value environmental problems and potential solutions (Innes and Booher, 2003; Brugnach and Ingram, 2012).

An increasingly recognized challenge in navigating these differences stems from differences in ways of knowing (WoKs). We conceptualize WoKs as socially embedded, relational systems through which individuals and groups generate, validate, and apply knowledge in order to interpret and engage with the world (Feldman et al., 2006). WoKs are not static bodies of information or synonymous with particular data sources; rather, they are ongoing, culturally and institutionally situated processes that shape how stakeholders frame problems, define what counts as credible evidence, and decide whose knowledge is considered legitimate (Feldman et al., 2006; Welsh, 2014). Analytically, we identify three dominant WoKs (local/experiential, scientific, and administrative) that emerged inductively from our data as broad types reflecting recurring epistemic orientations in how participants generated, validated, and applied knowledge in governance contexts.

In collaborative governance, WoKs are not just information but are relational and political, shaped by place, identity, values, social position, institutional role, and histories of conflict or collaboration (Gerlak and Brugnach, 2017; Almazán-Casali et al., 2021; Mukhtarov, 2015). These epistemic orientations influence how stakeholders frame problems, interpret evidence, assess trade-offs, and define priorities, thereby shaping visions of appropriate management and success (Feldman et al., 2006; Welsh, 2014). For example, a rancher may draw on experiential, place-based ways of knowing tied to seasonal land use and long-term observation, while an agency scientist may rely on monitoring data or models grounded in disciplinary norms. These differences do not constitute competing goals in and of themselves, but reflect how distinct WoKs shape interpretations of the landscape and inform judgments about desirable outcomes. Consistent with Feldman et al. (2006), these WoKs are not stable attributes but are continuously reproduced and reshaped through interaction within collaborative processes. As such, WoKs play a crucial role in shaping both the processes and outcomes of collaboration.

### Plural Ways of Knowing and Challenges in Collaboration

Although collaborative governance is intended to create an inclusive decision-making process, the integration of multiple WoKs is often shaped by existing power dynamics and institutional norms. Scientific and technical knowledge, which is often perceived as objective and generalizable, tends to be privileged over local or experiential knowledge, which may be dismissed as anecdotal or subjective (Morrison et al., 2019; Reed et al., 2018; Turnhout et al., 2012). This creates hierarchical structures within collaborative processes, which influence the degree to which WoKs are legitimized or marginalized. As a result, the process of integrating knowledge is not only technical but also political, as it reflects broader struggles over whose perspectives count in environmental decision-making (Gerlak and Mukhtarov, 2015; Almazán-Casali et al., 2021).

While integrating these different forms of knowledge can improve adaptive capacity and enhance governance legitimacy (Armitage, 2005; Tengö et al., 2014), doing so also introduces tensions around authority, participation, and equity. When one WoK is elevated above others, certain goals and success metrics may be privileged, while others are overlooked or discounted. Subsequently, attempts to integrate WoKs require careful consideration of the tensions that underlie the differences in understanding.

In conflicts related to natural resource management, differences in WoKs are commonly enmeshed in highly complex relationships, often involving power asymmetries (e.g., disparity of power between the resource manager and resource user), issues of identity (e.g., being a rancher vs. a federal employee), access or control over resources or information (e.g., allocation and distribution of permitted land uses), and know-how and expertise (e.g., knowledge about local practices vs. scientific method) (Schneider and Brugnach, 2017; Ingram, 2007). These complexities then impact whose voices and perspectives are legitimized in decision-making (Meijerink, 2005; Schneider and Ingram, 2007; Brisbois and de Morrison et al., 2019; Almazán-Casali et al., 2021; Loë, 2016). This understanding calls for greater attention not only to the presence of different WoKs within collaboration, but also to how stakeholders perceive, engage with, and use that knowledge in shaping collective goals and defining what success looks like. Based on this review of the literature, we ask the following questions:

- What types and sources of knowledge are present in the collaborative, and how do they inform different management goals?
- How do stakeholders perceive and engage with different ways of knowing in the context of collaboration?
- How do these perspectives on knowledge shape participants’ understandings of success in collaborative governance?

## Methods

### Thunder Basin Ecoregion Case Study

#### Study Area and Background

**Fig. 1:**
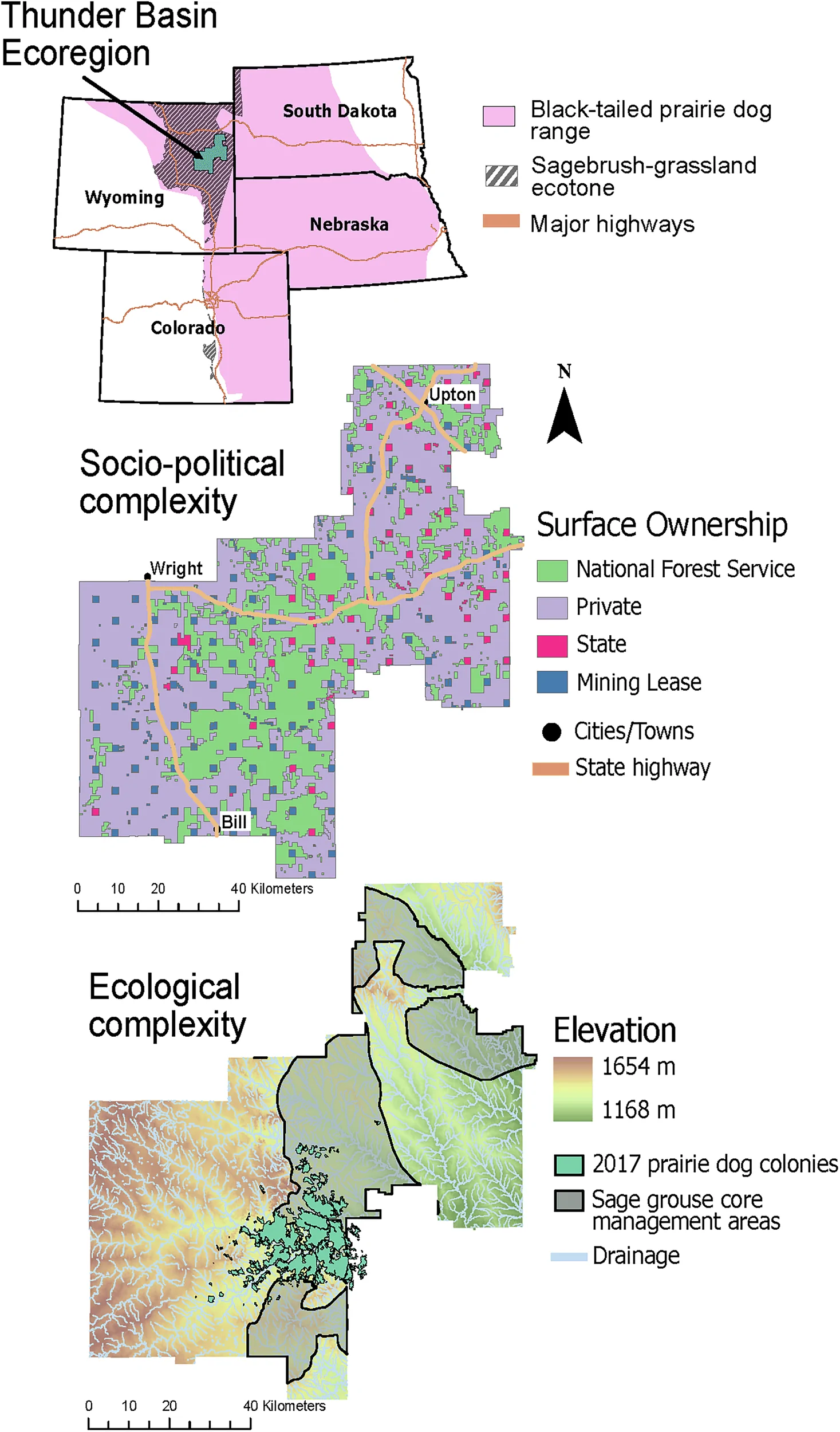
Thunder Basin Ecoregion in Wyoming, USA, is a dynamic social-ecological system influenced by intersecting ecological and socio-political factors. Maps by Erika Peirce; reproduced with permission (Wilmer et al., 2025)

This case study examines the Thunder Basin ecoregion (TBER), a 7000 km^2^ region located in northeastern Wyoming, USA (Fig. 1). TBER sits within a rangeland ecotone, or boundary zone, located between the Great Plains to the east and sagebrush steppe to the west (Porensky et al., 2018). The system supports numerous grassland and sagebrush species of conservation concern (Davidson et al., 2022; Duchardt et al., 2019; Duchardt et al., 2018), including black-tailed prairie dogs (*Cynomys ludovicianus*; hereafter: prairie dogs), mountain plovers (*Charadrius montanus*), and greater sage-grouse (*Centrocercus urophasianus*). Over the past two decades in TBER, prairie dog conservation efforts have also become a management priority for numerous conservation advocacy groups (Wilmer et al., 2025).

TBER also encompasses the Thunder Basin National Grassland, which is managed by the U.S. Forest Service (USFS) for multiple uses, including ranching, energy extraction, and wildlife conservation. These uses occur across a highly interspersed mixture of private and public lands, creating multiple intersecting land ownership and management interests. Ranching is central to the culture and economy of the region, with stocking rates and grazing seasons varying across allotments based on local conditions and management objectives. Management of federal and state lands permits grazing activities, and the overseeing agencies manage rangeland resources in collaboration with local grazing associations.

In recent history, conflict over management decisions in TBER has arisen as a result of extreme fluctuations, or “boom-bust cycles”, in prairie dog populations, which exacerbate differences between ranching and conservation goals (Connell et al., 2019a, b; Duchardt et al., 2019; Wilmer et al., 2019a). Specifically, populations of prairie dogs in TBER increased by 234% between 2012 and 2016, with the prairie dog complex exceeding 75,000 acres in size by 2017 (Davidson et al., 2022). However, from 2017 to 2018, a sylvatic plague event reduced prairie dog colony acreage by 99% (Davidson et al., 2022; Duchardt et al., 2023).

The resultant ecological and social upheaval highlighted differences in attitudes regarding prairie dog management. Specifically, black-tailed prairie dogs are classified as an agricultural pest by the state of Wyoming due to concerns about forage competition with livestock, alteration of vegetation structure, and potential impacts to grazing capacity and infrastructure (e.g., burrowing that may injure livestock or damage equipment). Conversely, prairie dogs are a keystone species and provide important habitat for other native species (Duchardt et al., 2019; Duchardt et al., 2018), and are classified as a species of special concern by the USFS. The resulting divergence in management goals created friction between different stakeholder groups, decreased trust among stakeholders, and strained the capacity of management agencies attempting to navigate these conflicting concerns (Wilmer et al., 2019a). Amidst the dramatic ecological shifts and discontent with existing management options, the conflict expanded beyond prairie dog management and came to represent a conflict over larger socio-political concepts such as equity, tradition, private property rights, government control, power, and acceptable forms of knowledge (Patterson et al., 2003).

In an attempt to respond to this conflict, a collaborative multi-stakeholder group known as the “TB working group” was formed to provide input to USFS in the preparation of an Environmental Impact Statement (EIS) to amend the Thunder Basin National Grassland Management Plan. However, in complex systems such as TBER, collaboration remains difficult due to the number of involved stakeholders and their diverse WoKs (Guerrero et al., 2015; Bodin, 2017), as detailed in the following section.

#### Thunder Basin Collaborative Working Group Efforts

Prairie dog management in TBER has been the focus of collaborative discussions for over 25 years, and in 1999 spurred the creation of a local non-profit, the Thunder Basin Grasslands Prairie Ecosystem Association (TBGPEA), a landowner-led organization focused on supporting science-based management that balances ecosystem conservation and agricultural productivity. In 2015, USFS approached the Ruckelshaus Institute at the University of Wyoming, an organization that facilitates collaborative natural resource decision-making, to assess stakeholder perspectives regarding prairie dog issues (USFS 2015). The assessment identified potential for a collaborative process to address management issues (Clement and Glendenning, 2015), leading to a series of public collaborative learning workshops in 2016–2018 that informed subsequent working groups.

The most recent effort, the TB working group, was convened in 2018 by the Wyoming Department of Agriculture and later the Wyoming County Commissioners Association to advise USFS on a Grassland Plan amendment revising prairie dog management approaches. The amendment’s scope was to balance prairie dog conservation and control with other grassland uses, in response to dramatic 2016–2018 population fluctuations that alarmed multiple stakeholders. Participants in the working group included representatives from federal management and research agencies, state and local agencies, non-profit organizations related to conservation and/or agriculture, academic and private researchers, and members from the local community, including livestock producers. In 2018, the group submitted its major set of recommendations to USFS and continued meeting through 2020.

USFS released the *Thunder Basin National Grassland Land and Resource Management Plan Amendment* at the end of 2020 (USFS 2020). The amendment was subsequently challenged in the courts by environmental conservation groups who did not participate in the working group, and in 2024, the 10th Circuit Court of Appeals remanded the case to USFS to address deficiencies in its analysis (*Western Watersheds Project* et al. *v. Vilsack*, et al. No. 23-8081, 10th Cir. Oct. 28, 2024; see D. Wyo. No. 1:22-CV-00214-SWS). As of 2026, the working group continues to meet, coordinate on-the-ground prairie dog monitoring, and provide management recommendations to USFS.

A comprehensive timeline of events relevant to this collaborative process is provided by Wilmer et al. 2025.

### Recruitment and Data Collection

Data was collected between 2018 and 2021 and was initiated at the invitation of the Thunder Basin Grasslands Prairie Ecosystem Association (TBGPEA). We worked with TBGPEA to identify and contact individuals who had been involved in management and/or decision-making processes in TBER. We initially used purposive sampling to recruit participants with a range of different backgrounds and areas of expertise (e.g., rancher, conservationist, scientist, agency official, etc.), in order to capture the diverse WoKs present in TBER management contexts. To ensure that a range of WoKs were represented in the study, we asked each participant if there were other relevant individuals we should contact. We continued interviewing new subjects through our referral process until we reached a point of diminishing returns, where no new information emerged with additional data collection (Lincoln and Guba, Morse, 2008; Wilmer and 1986; Fernández-Giménez, 2015).

We conducted 40 semi-structured interviews with 43 stakeholders^1^ (Table 1), 36 of which occurred in-person and 4 of which were conducted remotely over the phone during the COVID-19 pandemic. Roughly 20% of interviewees were producers, 50% represented state, federal, or local governmental agencies, and the final 30% represented NGOs, universities, or the private sector. One co-author participated in the study as an interviewee; consistent with ethical guidelines (World Medical Association 2013), they contributed to study design but did not analyze or interpret their own data.

**Table 1:** Summary table of participant type

Participant type	sub-type	*n* (%)	Description
Producer	*Cow-calf and/or sheep operator*	9 (21)	Individuals who self-identify as working ranchers and rely primarily on a livestock operation for their livelihood
Federal governments	*Management, research*	11 (26)	Federal entities tasked with managing, conserving, regulating, or researching natural resources under federal policy and authority
State governments	*Management*	5 (12)	State-level entities are responsible for the management, regulation, conservation, and monitoring of natural resources within state boundaries
County and local governments	*Management*	4 (9)	Local government bodies at the county, municipal, or community level are responsible for planning, managing, regulating, and monitoring local natural resources and related land-use decisions
NGO	*Agriculture, conservation, interdisciplinary*	6 (14)	Independent entities focused primarily on environmental, social, or policy issues related to natural resource management
University research	*Ecosystem science, rangeland ecology*	4 (9)	Individuals employed primarily by academic institutions, whose primary livelihood is derived from conducting original research, teaching, and disseminating knowledge
Private scientist / consultant	*Industry, consultant*	4 (9)	Professionals employed by private firms, consulting businesses, or operating independently, whose primary livelihood comes from providing expertise or technical support to clientele
Total		43 (100)	

These categories are used for communication purposes only and do not imply fixed boundaries, as some individuals may cross categories, and important variation exists within a participant type

Interviews ranged from 37 min to 3 h and took place at a location of the participants’ choosing. Interviews with producers often included conversations with multiple family members involved in the operation, as well as a ranch tour. When interviews included multiple family members from a single producer operation, these were treated as separate stakeholder units within a single interview for analytic purposes. Our interview guides inquired about management practices within TBER, relationships to TBER, sources of conflict, and how information is shared among stakeholders. A separate interview guide was utilized for ranchers and non-ranchers in order to reflect differences in experience and expertise. A more comprehensive summary of the interview guides is available in Supplementary Material A (Table A1), and full interview guides are available upon request. While the professional role provided a useful heuristic for identifying dominant epistemic orientations in this study, we recognize that WoKs are shaped by multiple, intersecting dimensions of identity, including length of residence, generational ties to place, age, and political or social positioning. Although participants often referenced these characteristics during interviews, they were not systematically collected as structured demographic variables and therefore were not analyzed comparatively. As such, occupational categories should not be interpreted as exhaustive or deterministic representations of epistemic orientation.

In addition to the interviews, the research team also conducted a focus group with TBGPEA board members in Fall 2019 to capture collective insights and group dynamics. Members of the TBGPEA board included ranchers, an environmental scientist affiliated with private industry, and the owner of a natural resource-based consulting firm. In 2021, the lead author engaged in participant observation with ranchers, USFS staff, and conservation NGO representatives to contextualize interview findings and observe interactions directly. Additionally, members of the research team either participated in or observed over 100 hours of collaborative working group meetings from 2019 to 2025. Finally, we reviewed relevant documents generated during the EIS development period, including scoping documents, public comments and responses, draft statements, amendments, and final records of decision.

Specific locations, names of individuals, and other identifying information have been omitted from the study. This study was conducted under Boise State University IRB protocol #10877.

### Data Analysis

We audio-recorded and transcribed all interviews to text. The lead author validated transcriptions for accuracy by comparing all transcripts against audio recordings, imported transcripts into qualitative data analysis software (NVivo), and documented analysis with a series of memos throughout the study (Tracy 2024).

To interpret stakeholder WoKs in relation to collaborative decision-making, the lead author engaged in an iterative examination of the WoK literature, collaborative governance literature, and our qualitative data. The lead author undertook multiple rounds of coding using an inductive-deductive approach that allowed themes to emerge both from the dataset and in relation to the theoretical framework informed by our literature review on knowledge and collaboration (Charmaz, 2006; Glaser and Strauss, 2017).

During initial coding, the lead author used an inductive approach to discover themes that arose from the data. Initial codes corresponded with the broad analytical questions used in the interview instrument: What do different actors make decisions about? What are the important sources of information for different actors, and how is information shared within and across groups? What are the successes and challenges of cross-group collaboration? What is driving conflict in TBER, and how is it being addressed? The results of initial coding were synthesized in a research memo and shared with members of the research team.

The lead author iteratively refined codes and developed a codebook that consisted of code names, descriptions, and examples (Tracy 2024). Subsequent rounds of coding focused on developing hierarchical codes by examining the transcripts line-by-line and labeling codes related to types and sources of knowledge of participants, management goals, perceptions and engagement with different WoKs during the collaborative process and how these perspectives shaped participants’ understandings of success in a collaborative environment. Through this iterative process, we identified recurring patterns in how participants described generating, validating, and applying knowledge across the collaborative. These patterns informed the inductive development of three analytic categories of ways of knowing (local/experiential, scientific, and administrative), which we use as heuristic constructs to interpret epistemic orientations rather than as fixed attributes of particular stakeholder groups. Our unit of analysis is participants’ articulated accounts and observed interactions, rather than individuals as fixed representatives of particular knowledge categories. We also recognize that participants’ positionality, including their professional roles, lived experience, and relationships to the landscape, shaped how they interpreted and engaged with different ways of knowing. Findings were also compared to relevant literature and triangulated from our focus group, participant observation, and document review.

To establish the trustworthiness of our qualitative findings, we applied the four criteria of credibility, transferability, dependability, and confirmability (Lincoln and Guba, 1986; Ahmed, 2024). Credibility was strengthened through prolonged engagement in the field ( > 100 h of meeting observation), triangulation of multiple data sources (interviews, focus group, participant observation, document review), and presentation of preliminary findings to participants and other stakeholders. Transferability was supported by providing a thick description of the collaborative context and extended quotations in Supplementary Material B. Dependability was addressed by following a consistent interview protocol, recruiting a representative sample across stakeholder categories, and maintaining a transparent audit trail through analytic memos and version-controlled codebooks. Confirmability was supported by documenting analytic decisions, using reflexive memos to acknowledge researcher perspectives, and grounding interpretations in direct evidence from the data.

## Results

This section presents findings related to the types and sources of knowledge present in collaborative contexts, and how different WoKs inform management goals. It then examines how stakeholders interact with different WoKs, and how these interactions shape their perspectives on collaboration and their understandings of success. Excerpts from the interviews are provided to illustrate how various WoKs are expressed and operationalized by participants. We present these WoKs as analytical constructs that capture recurring epistemic orientations in the data, rather than as fixed or mutually exclusive categories of participants.

This section provides a condensed summary of key themes; additional context, subgroup variation, and extended participant quotations are available in Supplementary Material B.

### What Types and Sources of Knowledge are Present in the Collaborative, and How Do They Inform Different Management Goals?

Participants described drawing on different WoKs that were shaped by their training, experience, and institutional roles. These sources of information (e.g., peer-reviewed literature, professional assessments, lived experience) are treated here as expressions of broader WoKs, rather than ways of knowing in themselves. While boundaries between WoKs were fluid, three general categories of WoKs (scientific, local/experiential, and administrative) were consistently referenced (Table 2). Extended descriptions of WoKs are available in Supplementary Material B.

**Table 2:** Summary of three generalized ways of knowing (WoKs) present in the Thunder Basin collaborative working group, including definitions, examples of use, mechanisms of knowledge verification, and illustrative quotes from interviews

Way of Knowing	Knowledge Production	Dynamics Over Time	Example of Use	Illustrative Quotes
**Administrative**	Interpretation and application of scientific, experiential, and legal information within institutional mandates; policy translation; procedural evaluation; coordination across agencies and stakeholders	Evolves with regulatory changes, institutional mandates, and shifting political and public accountability contexts	Balancing monitoring data, public input, legal requirements, and management feasibility to develop management plans	*“As far as the process and collaborative portion… we’re going to have to go through the entire NEPA process and objections and probably a lawsuit or two.” (TB27, state agency)*
**Local/Experiential**	Firsthand, place-based knowledge developed through daily experience, observation, trial-and-error, and generational exchange	Evolves through generational transfer, demonstrations of practical success, community networks of trust; cross-checking with agency and extension staff	Adjusting grazing based on forage availability and long-term observation; implementing drought responses rooted in lived experience	*“All I can tell you is years and years of being around ranching and just talking and being taught by my parents and my grandparents, and… just having an eye for what’s going to work and not work.” (TB02, producer)*
**Scientific**	Systematic observation, measurement, and experimentation; established methods to generate objective, generalizable results	Builds through iterative research, replication of studies and meta-analyses; evaluated by formal peer review processes and professional networks	Designing and interpreting ecological monitoring studies; publishing research on rangeland dynamics	*“What’s nice about replication is you can think about meta replication, just multiple studies over a large space, and those inferences, and someone could do a meta-analysis… and come up with the larger effect that may be out there.” (TB06, university research)*

Across all three WoKs, participants described learning and knowledge development as socially mediated processes. Whether through professional peer networks, inter-agency communication, informal exchange among colleagues, or intergenerational transfer within families and communities, knowledge was developed, interpreted, and reinforced within social relationships. While these processes differed in form and institutional context, they reflect parallel patterns of social learning operating within distinct epistemic communities rather than fundamentally different modes of knowledge formation. In the sections below, we describe how participants articulated these WoKs across different roles and contexts, recognizing that individuals often drew on multiple WoKs rather than fitting neatly into a single category.

#### Types and Sources of Knowledge

Scientific WoKs emphasize empirical observation, reproducibility, and peer review to produce objective, generalizable knowledge. Participants in university and federal research roles described drawing on scientific WoKs characterized by peer-reviewed and gray literature, as well as collaborative research processes, as central to their practice. Participants from NGO and agency contexts described using scientific WoKs mainly for ecosystem monitoring and regulatory enforcement. At state and local levels, respondents described relying on internal professional networks and trusted colleagues as key channels through which scientific and policy-relevant information was interpreted and applied.

Local/experiential WoKs rely on firsthand, place-based knowledge developed through daily experience, observation, trial-and-error, and generational knowledge transfer. Participants with long-term, place-based ties to the region, including ranchers, emphasized local/experiential WoKs through practical application. Although the length of residence was not systematically measured, several participants emphasized generational or long-standing relationships to the landscape as central to how they understood and interpreted management issues. Participants also collected informal longitudinal data (e.g., wildlife monitoring), shared insights within community networks, and collaborated with extension agents and agency staff.

Administrative WoKs are grounded in how individuals interpret, evaluate, and operationalize knowledge within institutional and regulatory contexts. While agency staff and affiliated actors draw on scientific information and professional experience, administrative WoKs are distinguished by how knowledge is assessed for credibility, relevance, and usability under statutory requirements, policy constraints, and accountability to multiple audiences. Rather than producing new empirical knowledge, administrative WoKs reflect an orientation that emphasizes the interpretation, integration, and translation of diverse forms of evidence into forms that are considered actionable and defensible within governance processes, often generating new forms of policy-relevant knowledge through this process.

Participants described drawing on scientific assessments, local and experiential knowledge, professional judgment, and public input, while evaluating how these sources align with legal mandates and institutional responsibilities. Local agency staff emphasized contextualizing formal regulations within place-based understandings, while NGO staff and private consultants described mobilizing administrative knowledge to navigate regulatory processes, support compliance, and facilitate communication across stakeholders. Across these accounts, administrative WoKs were characterized less by a distinctive source of information than by a mode of reasoning oriented toward evaluating, integrating, and legitimizing knowledge within governance contexts. In this process, knowledge is not only applied but actively interpreted, translated, and rendered actionable, such that administrative practice shapes how knowledge is defined, prioritized, and used in decision-making.

#### Ways of Knowing Shape How Management Goals are Defined

Stakeholders across groups described a shared goal of achieving balance in land management. However, interpretations of balance varied, shaped by differences in WoKs as well as institutional roles, land tenure, or organizational mandates. While management goals themselves do not constitute distinct WoKs, participants’ goals reflected how different epistemic orientations shaped problem framing, interpretations of evidence, and assessments of trade-offs. Taken together, these differences in management goals do not reflect discrete or incompatible objectives but rather illustrate how distinct WoKs shape how stakeholders interpret ecological conditions, prioritize concerns, and define what successful management looks like.

##### Local Knowledge and Management Goals.

Participants engaged in ranching livelihoods emphasized balancing economic viability with long-term land stewardship. Their goals reflected site-specific knowledge of the landscape and a desire to sustain the range for future generations. As one participant described, “My goal is just to make it as functional [sic] and make it better… anything I can do to help the range, help the water, the wildlife, all of it… just trying to improve what’s here, and sustain a living” (TB02, producer). While many valued conservation outcomes, others voiced concerns about management approaches (such as protections for prairie dogs) that felt misaligned with the viability of working lands.

##### Scientific Knowledge and Management Goals.

Participants drawing on scientific WoKs often emphasize ecosystem processes and landscape-scale outcomes that necessitated trade-offs and synergies across multiple land uses. One researcher explained, “These are lands that are… managed for multiple ecosystem services. Most of my research has been trying to address trade-offs or synergies in… achieving those services” (TB10, federal research). Others described how engaging with local WoKs deepened their understanding or shaped new lines of inquiry. One scientist reflected that, “Because of what [local producers] asked questions about, we now have a much more robust explanation of what went on” (TB09, federal research), describing how incorporating stakeholder perspectives sometimes prompted them to revisit and broaden earlier interpretations of data.

##### Administrative Knowledge and Management Goals.

Participants in agency roles drawing on administrative WoKs described management goals in relation to navigating between technical information and administrative mandates, while also attending to community concerns. Many described cross-agency information sharing through informal reports and communications, as summarized by one local official: “I go to workshops, and I talk to anyone with expertise. I talk to Game & Fish and Fish & Wildlife; I talk to the Prairie Dog Coalition… I find people with expertise in the area” (TB40, county agency). Federal employees also described a goal of “walking that balance” in regard to finding a “sweet spot” between ecological goals and public acceptance, for example, maintaining viable prairie dog populations while keeping colonies off key cattle ranges to reduce conflict. Many officials reside in the surrounding community and emphasized wanting to build trust and understanding at the local level. As one participant described it, “When you’re dealing with someone’s livelihood, you can’t always just consider the science. There has to be that balance between that cultural need and that scientific need” (TB16, federal management).

### How do Stakeholders Perceive and Engage with Different Ways of Knowing in the Context of Collaboration?

**Fig. 2:**
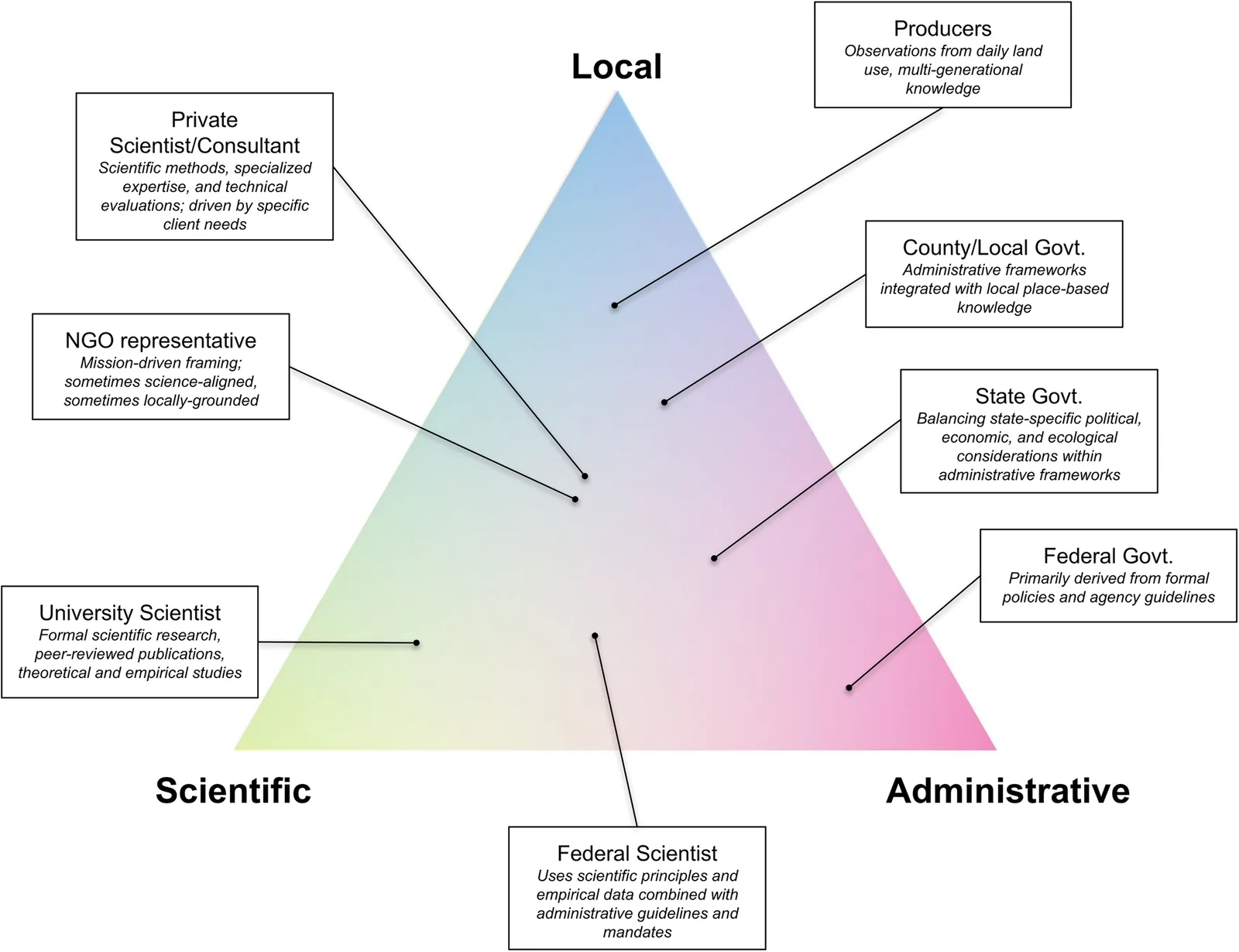
Approximate positioning of stakeholder groups along three overlapping ways of knowing (WoKs): local, scientific, and administrative. Federal categories distinguish between research-focused scientific roles and management or regulatory roles within federal agencies. Positions represent tendencies rather than fixed categories: there is significant variation within stakeholder groups, and individual participants often draw on multiple WoKs depending on context

Collaborative processes provided stakeholders opportunities to engage with diverse WoKs, yet also surfaced tensions regarding their perceived legitimacy. These perceptions varied across and within stakeholder groups and suggest that perceptions are shaped not just by roles, but by individual experiences, values, and relationships (Fig. 2). Interactions between WoKs are described below, while a more detailed exploration of how stakeholders describe and navigate different WoKs is provided in Supplementary Material B.

#### When Ways of Knowing Collide

Tensions between different WoKs emerged most visibly when scientific findings contradicted stakeholders’ lived experiences. For example, participants frequently cited a recent study suggesting prairie dog colonies may enhance forage quality under certain conditions (Connell et al., 2019b). Conservation-oriented respondents felt the findings reinforced their understanding of prairie dogs’ ecological role. As one participant explained, “At certain levels… colonies will actually increase the nutritional value of the vegetation that is on their colonies… There may be less available forage for cattle, but every bite they take is more efficient, and more nutritious” (TB11, conservation NGO).

Conversely, several producers rejected these conclusions as disconnected from their observed realities, with one producer dismissing the study as “hogwash” because the findings contradicted the visibly barren conditions experienced firsthand: “She had a big research paper and said, “There’s more [grass] production on prairie dog towns because they chop it off and so it’s higher protein…” I mean, what she’s saying may be true, but [the forage] is all that tall [makes hand gesture denoting very short]” (TB21, producer).

Other participants acknowledged a mismatch between the scale of scientific observation and stakeholders’ experience, explaining that the measurements were taken on small colonies at the edges of the complex, while many landowners were dealing with large colonies where “there was just absolutely no forage” (TB10, federal researcher). While not disputing the study’s methods or findings, the researcher acknowledged that the study’s credibility was compromised by underlying issues: the science wasn’t “wrong”, but it wasn’t addressing the “right” question regarding stakeholder concerns (i.e., visibly barren landscapes).

#### Learning Across Boundaries

Despite tensions, participants described meaningful instances of mutual learning across WoKs. Producers’ observations prompted new scientific questions, scientists reframed data through local insight, and agency staff worked to reconcile top-down mandates with place-based knowledge. One federal researcher explained how encountering experiential knowledge prompted deeper inquiry: “If a rancher tells me something surprising… there’s a reason for that, right? … It makes me want to go bigger or go in a different direction… It just makes me think maybe I missed something else that’s going on.” TB13 (federal research). Agency staff also emphasized blending short-term scientific findings with long-term, place-based observations, recognizing the value of multi-generational knowledge in interpreting local trends in order to address “what’s missing”. As one federal manager stated, “You can’t just come and do research for two or three years and answer a question that may have taken 50 years, 70 years of watching the landscape change and get an answer, and so sometimes that cultural aspect, that history within families is so vitally important” (TB16, federal management).

Participants also described how long-term collaboration (over the course of 20+ years) shifted perspectives on the relevance of research, particularly when researchers engaged directly with managers to identify knowledge gaps and management needs. One agency representative described this transition, stating:

“I was not a fan of research, having not had researchers ever really ask what management needed. And then they come in with this data, and it’s like… that’s not helpful. Then they would walk away with the data, too, a lot of times.

That was one thing that was really neat… for a long time now, we’ve been able to meet with the researchers and talk, and that’s what they ask. “What do you need?” They take our feedback… so now I’m a big fan.” - TB24 (federal management)

While epistemic tensions persisted overall, participants were able to identify conditions under which integration was possible. In such cases, shared inquiry served as a catalyst for continued dialogue. Stakeholders characterized successful knowledge integration as a product of transparent research processes, openness to feedback, mutual respect, and sustained engagement across multiple WoKs. Participants’ accounts suggest that shared inquiry was not only a matter of exchanging information but was grounded in dispositions such as openness to alternative perspectives, recognition of the limits of one’s own knowledge, and a willingness to engage across differences in goals and values. In several cases, participants emphasized that moving beyond entrenched positions required shifting from defending knowledge claims toward collaboratively exploring uncertainty and trade-offs. As one county manager summarized, effective collaboration required “professionalism from all corners… if everybody will give a little, you can go a long way… You need every bit of that from every corner” (TB40, county management; see Supplementary Material B for further details and extended quotes).

### How do Perspectives on Knowledge Shape Participants’ Understandings of Success in Collaborative Governance?

Participants emphasized the central role of knowledge in shaping their experiences with collaboration. Rather than treating knowledge as neutral, many described how it was selectively interpreted or strategically used by stakeholders, influencing both how participants engaged with the process and assessed outcomes.

#### Selective Interpretation and Strategic Use of Knowledge

Participants described, and observations of collaborative interactions also indicated, their perceptions that both scientific and experiential WoKs were sometimes selectively referenced to support particular management goals. Instead of treating knowledge as static or universally applicable, respondents reflected on how different actors appeared to frame evidence in ways that aligned with their own perspectives or desired outcomes. Selective use of knowledge was particularly evident in discussions around controversial species like prairie dogs and their impacts on wildlife and rangeland health. Describing how individuals used different WoKs to support competing claims, one state agency official described:

“Somebody comes in and says, ‘Well, we know from studies that prairie dogs only do this.’ Then you have somebody else over here saying, ‘Well, but from my experience as the owner of those lands for 100 years, I could tell you that’s not totally true.’” - TB25 (state agency)

In this context, scientific and experiential claims were both presented as authoritative despite divergent conclusions. The same respondent noted that the perceived value of a study often depended less on its methodology and more on its alignment with stakeholder preferences: “You have all those figures, and of course, in a situation with sides, and with desired outcomes, people are going to try and use those to fit their situation.”

Others observed that knowledge is “situated,” meaning that it is developed and actionable in certain contexts (Schwandt, 2000; Robbins, 2006; Wilmer et al., 2018), and therefore stakeholder positions could influence how research questions were framed. As one county agency representative explained:

“I think [the research] is reliable. But it’s just like anything. If you are for expansion, that’s where you’re going to lean your research toward. If I were out there doing research on the damage, it would go the other way. So I think it all depends on what side of the line you’re on.” -TB40 (county agency)

Participants also reflected on the influence of selective knowledge use on collaborative discussions, especially when knowledge was used to assert authority rather than invite dialogue. One state agency respondent described a contentious public exchange over mountain plovers:

“When people try to weaponize whatever knowledge they think they have, that’s when you get into a bind… Somebody who got up and said, “Oh I’ve seen plovers in tall grass. It’s not like they don’t use it,” and somebody else got up and said, “You’re full of crap. You don’t know what you’re talking about and I’m a scientist’ or whatever. That’s where you do have a problem with that, as opposed to if the question is ‘I’m pretty sure I’ve seen a plover in tall grass. What do you know about that,” and they go, “Don’t know. I’ve never seen it. We’ve never studied it though so maybe it’s possible.” That’s much different because you’re bringing two pieces of knowledge together there to try and actually have that conversation rather than “I know this” and “I know this.”” -TB27 (state agency)

Here, scientific knowledge was used to invalidate rather than explore alternative perspectives. The hypothetical second response, however, could have created space for mutual learning by acknowledging the limitations of both scientific and experiential WoKs. Participants also linked selective use of knowledge to historical tensions around whose knowledge was legitimized. A federal manager noted that repeated dismissal of local observations influenced how landowners engaged with ecological research: “It doesn’t matter what research says… and it may be just because they’ve gotten hammered on all these years. Every time [landowner] said, ‘We’ve seen them [wildlife] somewhere else,’ somebody said, ‘You’re wrong.’” (TB24, federal management). Such interactions contribute to a pattern in which landowners rely more heavily on personal or place-based knowledge, particularly when they feel their observations have been consistently challenged or discredited. Consequently, scientific findings were sometimes viewed as less trustworthy, especially when they do not align with firsthand experience, as described above. While these perceptions were often rooted in local histories of conflict, they also reflect broader dynamics shaping trust in scientific institutions in contemporary environmental governance contexts. In this way, skepticism toward science within the collaborative cannot be understood solely as interpersonal disagreement, but as embedded within wider debates about expertise, authority, and legitimacy.

These accounts, alongside observations of collaborative interactions, suggest that selective interpretation and strategic use of knowledge were both experienced and enacted within the collaborative process. While our analysis does not seek to adjudicate the validity of particular knowledge claims, it highlights how both observed dynamics and participants’ perceptions of selective use shaped trust, legitimacy, and engagement within the collaborative setting. These dynamics were central to how participants navigated epistemic differences and evaluated the success of collaboration. In some cases, this reflected differences in how participants engaged with knowledge itself: whether as a means of understanding ecological conditions, or as a resource for advancing particular management priorities.

#### Perceptions of Woks Influence Attitudes Toward Collaboration

Interviews revealed a strong association between participants’ perceptions of WoKs and broader attitudes toward collaboration. Respondents who valued multiple WoKs and were open to cross-group learning often described collaboration positively, seeing it as an opportunity to bridge gaps and co-create solutions. One conservation NGO representative highlighted collaborative potential:

“How can we reach out to the people that have a lot to say in all of the silos of categories of people that we’re working with to find out how to move forward now?… We have an opportunity. What are the steps to take? What are the barriers, and how can we move forward? What are the needs? Forest Service still has to manage prairie dogs, landowners still live on the ground, and we still want ferrets. Okay, so let’s come up with a game plan, and can we organize a group of people to create that game plan.” -TB20 (conservation NGO)

This illustrates how bringing stakeholders together prompted individuals to consider how their expertise could advance broader group goals rather than remaining entrenched in individual siloes. Others described a shift among participants in how they approached collaborative discussions. One respondent recalled observing other participants broadening their framing of land management to consider diverse stakeholder interests: “They’ve [producers] become engaged in a productive way that’s not just, how do we maximize production? It is, how do we sustain ecosystems and livelihoods in this region?” (TB13, federal researcher).

However, not all participants shared this optimism. Some, particularly those representing scientific or conservation-focused perspectives, expressed dissatisfaction when they perceived their contributions as undervalued and described feelings of exclusion and reduced confidence in the value of participating. One university-affiliated researcher recalled their experience in collaborative meetings as discouraging due to the perceived lack of appreciation for scientific input: “Scientists’ voices should matter regardless, and so should anybody from the public. I don’t feel like that’s being appreciated or respected or included, and so therefore… I was very disturbed by the meetings” (TB18, university research).

Others shared frustrations about exclusion from discussions, particularly around wildlife conservation issues. Some respondents (often holding positions they perceived as outside the dominant consensus) expressed concern that their perspectives were not given equal weight, and that this imbalance undermined the integrity of the process. These individuals also described feelings of weariness and questioned whether the collaborative process was truly inclusive or capable of addressing the full range of concerns:

“We’re forbidden from discussing some of these realities… if we dare try to bring it up, we are told that’s off the table… We’re bullied basically into being quiet and complying with the other side’s desire to talk about how to get rid of the planned protections. Meanwhile, we have an environmental disaster occurring on the landscape, and nobody will even allow us to discuss it.” -TB11 (conservation NGO)

At the same time, other participants felt that discussions were often dominated by the most vocal stakeholders, which could crowd out more moderate or conciliatory perspectives, creating an environment in which both “outside” and “middle ground” participants felt sidelined for different reasons. One federal agency representative reflected on this imbalance:

“The people that are … really willing to compromise aren’t the loudest… Oh, you know, the old expression, ‘The squeaky wheel is the one that gets greased’? And that’s kind of how it feels… the ones that are making the most noise are the ones that get addressed, and yet we have a huge, like 98% of the folks that we deal with are not outlying groups, and they really aren’t so polarized. They’re much more, where they can kind of come to the middle and work together. But we have a few distinct folks on each side that make it very, very, very polarized.” - TB16 (federal management)

These accounts illustrate how perceptions of whose knowledge was recognized as credible and legitimate shaped attitudes toward collaboration, influencing whether they engaged in good faith, withdrew from the process, or viewed outcomes as meaningful and acceptable. While some remained hopeful about the potential for inclusive decision-making and shared solutions, others reported adversarial dynamics when they felt particular WoKs were dismissed. These varied experiences reflect the complexity of multi-stakeholder collaborations and provide the basis for further interpretation in the discussion that follows.

## Discussion

We sought to examine how stakeholders in TBER draw on different WoKs in collaborative land management, focusing on how knowledge diversity shapes management goals, perceptions of credibility, and attitudes toward collaboration. While participants demonstrated the capacity to engage with multiple forms of knowledge, WoKs were often applied in uneven and context-dependent ways. We observed that perceptions of knowledge were closely tied to broader understandings of management priorities and collaborative dynamics.

Our findings suggest that interactions between WoKs are shaped by institutional structures, historical relationships, and power dynamics. By examining how participants themselves interpret and navigate epistemic tensions, this study extends collaborative governance scholarship beyond structural analyses of participation and institutional design to foreground the relational and interpretive dimensions of knowledge use. Rather than treating knowledge integration as a technical challenge, our findings highlight its political and social character, shaped by histories of legitimacy, institutional mandates, and identity.

While findings from this case are not intended to be broadly generalizable, they offer insight into how stakeholders experience, negotiate, and mobilize different ways of knowing within collaborative governance processes. In doing so, they demonstrate how epistemic dynamics shape interaction, learning, and decision-making within collaborative governance processes.

### Ways of Knowing Shape Management Goals, Learning, and Knowledge Integration

Collaborative governance in complex socio-ecological systems is shaped not only by stakeholder diversity but also by the interplay of multiple WoKs. In TBER, the convergence of scientific, experiential, and administrative knowledge gave rise to both tensions and opportunities for mutual learning. Our findings indicate that knowledge is not merely integrated or rejected, but actively co-produced through interactions among stakeholders, institutions, and governance processes. Consistent with STS scholarship, participants evaluated knowledge claims not solely on their perceived accuracy, but also on their legitimacy, relevance, and alignment with existing relationships and management priorities (Boshoff, 2014; Brugnach, 2017; Jasanoff, 1987; Nightingale, 2016). However, our findings suggest that these dynamics extend beyond knowledge production. Participants also evaluated the credibility of different WoKs through their perceptions of who was advancing particular claims, their institutional affiliations, and their roles within the collaborative process. In this way, the case illustrates how negotiations over knowledge are simultaneously negotiations over legitimacy, authority, and belonging within collaborative governance.

These dynamics underscore the central role of social learning in collaborative governance. Prior research has emphasized collaborative processes as spaces of mutual adaptation and joint problem framing (Innes and Booher, 2003; Armitage et al., 2011; Reed et al., 2018). However, our findings suggest that learning across WoKs wasn’t automatic nor evenly distributed. Rather, participants’ willingness to engage with alternative forms of knowledge depended on trust, prior relationships, perceptions of legitimacy, and institutional flexibility. Consistent with Emerson et al. (2012) and Wyborn et al. (2019), these findings highlight the importance of creating collaborative spaces that support meaningful epistemic exchange. At the same time, they suggest that stakeholder diversity alone may be insufficient to facilitate learning unless collaborative processes also cultivate the relational conditions necessary for participants to critically engage with and learn from different ways of knowing.

Our findings also highlight the distinct role of administrative WoKs within collaborative governance. While scientific and local WoKs were often discussed as competing sources of information, administrative WoKs functioned as interpretive frameworks through which knowledge was translated into actionable management decisions. This finding suggests that administrative WoKs may play a mediating role in collaboration, shaping not only how information is evaluated but also how different forms of knowledge become institutionally relevant and actionable. In doing so, our results extend discussions of knowledge integration by highlighting the often-overlooked influence of administrative and procedural forms of expertise in structuring collaborative decision-making.

#### Politics of Knowledge Integration

Building on these dynamics of relational learning, our findings also reveal how the politics of knowledge integration shaped which knowledge claims gained traction within the collaborative. Although scientific WoKs are often regarded as the “gold standard” of knowledge in environmental policy contexts due to their perceived objectivity and rigor, our findings suggest that in TBER, this assumption is frequently contested. Producers and other local actors whose experiential knowledge is grounded in generational ties to the land often challenged the authority of scientific claims when they conflicted with lived realities. Rather than viewing scientific knowledge as universally privileged, participants deployed different WoKs strategically, often in alignment with their personal objectives or institutional affiliations. This pattern of use suggests that knowledge use and reception are deeply shaped by positionality and historical relationships. Scientific findings were not always accepted as neutral; instead, they were often politicized and interpreted through the lens of stakeholders’ values, goals, and social positioning (Boshoff, 2014; Gerlak and Morrison et al., 2017; Almazán-Casali et al., 2021; Mukhtarov, 2015; Turnhout et al., 2012). These findings extend STS scholarship by illustrating how credibility and authority are not intrinsic properties of scientific knowledge, but are socially constructed through processes of boundary work, institutional endorsement, and public trust (Gieryn, 1999; Jasanoff, 1987; Sarewitz, 2004).

At the same time, skepticism toward scientific knowledge in TBER cannot be understood solely as a reaction to local dismissal. Participants’ reflections suggest that broader political and cultural currents shaping trust in scientific institutions also informed how research findings were received. As a result, epistemic tensions within the collaborative reflected not only localized histories of conflict but also wider debates about expertise, authority, and legitimacy in environmental governance (Sarewitz, 2004). Our findings illustrate how these broader debates become embedded within local collaborative processes, shaping how stakeholders interpret and respond to scientific knowledge in practice.

In contrast, we observed moments where knowledge was used pragmatically, rather than ideologically. While some participants appeared primarily concerned with affirming the authority of their own WoK, some were willing to draw selectively on any WoK that supported their goals, whereas others demonstrated openness to using any form of knowledge that might support problem-solving and compromise (Supplementary Fig. C1). The distinction was evident in discussions where entrenched positions led to impasse, and more flexible participants were able to bridge divides in knowledge perspectives and move conversations forward. Rather than acting as formal ‘knowledge brokers,’ these individuals exhibited a practical orientation toward knowledge, treating it as a tool for governance rather than a marker of identity. This finding complicates conventional models of knowledge brokering that emphasize designated intermediaries (Bednarek et al., 2018; Meyer, 2010), suggesting that integrative epistemic work may emerge informally through relational practice and situational reflexivity. As described earlier, two federal employees independently offered examples of this integrative approach when describing moments of asking “What’s missing?” when confronted with divergent knowledge claims. In both cases, knowledge was approached as a tool for shared problem-solving rather than a fixed credential or identity claim. In TBER, these types of productive exchanges were made possible in part by the collaborative partnerships built by TBGPEA through the 2000s, as well as the extended period of collaborative learning that preceded the collaborative working group. These early efforts laid the groundwork for conditions conducive to cross-boundary learning and adaptive management, such as building long-standing relationships, iterative engagement, and mutual respect (Tengö et al., 2014; Wyborn et al., 2019).

Our findings also suggest that the legitimacy of knowledge claims was shaped by their alignment with stakeholder values, lived experience, and existing relationships of trust. Agency actors occupied a particularly influential position in this process because they were responsible for translating diverse forms of knowledge into management decisions. As a result, the credibility and actionability of knowledge were negotiated through ongoing social and institutional interactions rather than derived from evidence alone. These findings illustrate how questions of knowledge are often inseparable from questions of legitimacy and authority within collaborative governance processes.

### Conflict in and Between Ways of Knowing

Conflict among WoKs was common in TBER, but participants did not view conflict as uniformly negative. While entrenched commitments to particular knowledge systems sometimes contributed to frustration, fatigue, or stalemate, participants also described instances in which disagreement prompted reflection, learning, and creative problem-solving. Consistent with deliberative governance theory, these findings suggest that conflict can serve as a mechanism for surfacing underlying assumptions and values rather than simply an obstacle to collaboration (Dryzek, 2000; Innes and Booher, 2010).

Importantly, many conflicts were not solely disagreements over information. Participants frequently linked particular WoKs to identities, livelihoods, institutional roles, and experiences on the landscape. As a result, disputes over knowledge claims reflected deeper questions about credibility, legitimacy, and whose perspectives should shape management decisions. This finding extends scholarship that frames conflict as a productive force in governance by illustrating how epistemic conflicts are often inseparable from broader social and relational dynamics (Aylett, 2010; Mancilla García et al., 2019).

These findings suggest that supporting knowledge integration requires more than simply bringing diverse stakeholders together. While participants often expressed appreciation for knowledge diversity in principle, collaboration was frequently approached as a venue for asserting expertise or defending existing positions. Individuals who demonstrated openness to learning across perspectives were better positioned to facilitate integration, whereas those focused on validating their own views tended to reproduce knowledge hierarchies. In this sense, productive engagement across WoKs depended not only on the diversity of perspectives represented but also on the relational conditions that supported humility, trust, and mutual learning (Brugnach, 2017; Porensky, 2021; Wyborn et al., 2019).

#### Ways of Knowing Shape Perceptions of Collaboration

Stakeholders’ perceptions of success in collaborative governance were closely tied to their WoKs. In TBER, participants held different visions of what constituted effective land management, informed by epistemological orientations grounded in scientific research, professional mandates, or place-based experience. These divergent WoKs shaped how individuals defined management goals and evaluated the fairness and effectiveness of collaborative proceedings.

These findings reinforce the idea that collaborative governance cannot be understood solely in terms of outputs or formal agreements. In TBER, navigating competing goals and overlapping priorities required ongoing negotiation over what constituted credible knowledge, legitimate participation, and desirable management outcomes. This suggests that stakeholders’ perceptions of collaborative success were shaped by whether the process recognized and accommodated the WoKs they considered most relevant. Consistent with scholarship emphasizing collaborative governance as a process of joint meaning-making and negotiating legitimacy (Innes and Booher, 2003; Cleaver and Whaley, 2018; Westerberg et al., 2025), our findings demonstrate how different ways of knowing shape not only management preferences but also how collaborative outcomes are evaluated.

These findings also point to the importance of continuity in shaping how WoKs are developed, trusted, and applied within collaborative governance. Participants frequently described long-standing, and in some cases intergenerational, relationships to both the landscape and to one another, suggesting that the credibility and legitimacy of knowledge claims are built through repeated engagement over time. In this context, continuity in participation and institutional memory may play an important role in sustaining relationships across management cycles and maintaining the contextual knowledge necessary for integrating multiple WoKs. This observation may be particularly relevant in settings characterized by frequent agency staff turnover, where disruptions in relationships and local knowledge can complicate efforts to build trust and sustain collaborative learning (Innes and Booher, 2010; Wyborn et al., 2019). Recent research similarly suggests that frequent staff turnover and short-term assignments in federal agencies can hinder the development of locally-embedded expertise and erode trust in collaborative governance processes (Charnley et al., 2025; Puntenney et al., 2025), a challenge that is likely to only be exacerbated by existing conflict.

### Limitations

While this case offers important insights into the relational dynamics of collaborative governance, its findings are bound by the unique political and cultural context of TBER. Stakeholders themselves frequently described the region as distinct, and our results may not generalize to other settings with different institutional arrangements or histories. Additionally, our methodology may be limited by selection bias in participant recruitment and by the interpretive lens of the researchers. While our analysis focuses on participants’ professional roles as a key lens through which ways of knowing were expressed and interpreted, we recognize that other dimensions of identity, such as length of residence, political orientation, or socioeconomic background, may also shape epistemic perspectives. These factors were not systematically captured in our data collection and represent an important area for future research.

We also note the absence of Indigenous or Tribal perspectives in this study. While Indigenous knowledge systems are widely recognized as critical to natural resource governance, Tribal Nations were not represented among participants in this particular collaborative process. As such, our analysis reflects the perspectives of stakeholders engaged in this specific institutional context rather than the full range of knowledge systems relevant to the region. This absence reflects the composition and scope of the collaborative itself, rather than a determination that Indigenous knowledge is not relevant to rangeland governance. Future research should more directly examine how Indigenous knowledge systems and Tribal governance intersect with collaborative natural resource management in this and similar contexts.

### Future Research

Future research should pursue longitudinal and comparative studies that examine how trust, knowledge integration, and conflict transformation evolve over time. Emerging scholarship has begun to explore how place-based attachments, identity, and power shape collaborative governance processes (Leith et al., 2014; van Duncan, 2016; Kerkhoff, 2014; Wilmer et al., 2019b). Building on this work, future research could examine how these dynamics influence the legitimacy of different WoKs and shape stakeholders’ perceptions of fairness, belonging, and influence. Such research will further clarify how structural and sociocultural conditions interact to shape environmental governance outcomes and inform governance strategies that are more responsive to the cultural and emotional dimensions of contested landscapes.

## Conclusions

This study investigated how diverse ways of knowing (WoKs) shape collaborative natural resource governance in a complex social-ecological system. Through interviews, participant observation, and document analysis, we examined how different WoKs informed stakeholders’ perceptions of management goals, knowledge legitimacy, and participants’ perceptions of collaboration in the Thunder Basin ecoregion (TBER). Our findings reveal that knowledge diversity plays a central role in shaping collaborative processes and outcomes. Stakeholders brought scientific, experiential, and administrative forms of knowledge into dialogue, but the degree to which these WoKs were recognized, respected, and integrated varied considerably. Collaborative governance in TBER was not only a matter of assembling diverse participants, but also a process in which power dynamics, historical relationships, and institutional cultures shaped whose knowledge counted, for whom, and under what conditions.

These insights extend collaborative governance scholarship by demonstrating that differences in WoKs reflect broader differences in how stakeholders define management goals, evaluate legitimacy, and interpret collaborative processes (Brugnach, 2017; Nightingale, 2016; Turnhout et al., 2012). Our findings suggest that fostering knowledge integration requires more than bringing diverse stakeholders together; it also requires creating the relational conditions that enable participants to critically engage with and learn from different WoKs.

This expanded conceptualization is especially relevant in other contexts characterized by: (1) multiple, potentially conflicting ways of knowing (such as Indigenous, scientific, and local ecological knowledge); (2) diverse stakeholder groups with different relationships to place and resources; (3) complex social-ecological systems where technical solutions alone are insufficient; and (4) the need for ongoing governance and adaptive management capable of navigating evolving trade-offs, risks, and uncertainties over time. For example, marine protected area planning often requires integrating scientific data with fishers’ experiential knowledge and community values; watershed management requires coordination between producers, conservationists, and regulators; and climate adaptation planning increasingly depends upon bridging scientific projections, local observations, and traditional ecological knowledge (Agrawal and Armitage et al., 2011; Cinner et al., 2012; Gibson, 1999). In each of these settings, as in TBER, effective governance is contingent upon the interpretation, sharing, and negotiation of knowledge in pursuit of collective action.

Finally, our findings also carry important implications for practice. Inclusive collaborative processes must go beyond representational diversity to invest in the relational work necessary for meaningful dialogue across WoKs. This includes cultivating long-term trust, structuring opportunities for reflection, and designing facilitation approaches that recognize difference without forcing premature consensus. Practitioners should also be mindful of how organizational norms and institutional procedures shape the perceived legitimacy and usability of different knowledge forms. At the same time, our findings underscore that collaboration also poses emotional and ethical challenges. For many participants, the politics of knowledge felt deeply personal and consequential, leading to disputes that at times appear intractable. Participants’ accounts suggest that successful collaboration often depended less on resolving fundamental disagreements and more on participants’ willingness to engage with care, acknowledge others’ knowledge in good faith, and set aside the impulse to be “right.” Alongside formal structures and technical solutions, fostering epistemic humility, cultivating shared norms of engagement, and supporting boundary-spanning leadership may be equally essential to effective governance outcomes.

## Supplementary information


Levy_SupplementaryMaterial_EM


## Data Availability

Per the requirements of our human subjects research protocol (evaluated at Boise State University), raw data are not available to the public, but de-identified, summarized data are available upon request to the corresponding author.

## Footnotes

1 This sample represents a substantial proportion of the total stakeholder population: working group meetings typically draw 15–25 participants, and fewer than 100 individuals regularly engage in collaborative or management activities across the Thunder Basin region.
