# Supplementary material for: Contesting and Integrating Multiple Ways of Knowing in Collaborative Natural Resource Governance
Source: Environ Manage. 2026 Jul 22;76(8):256. doi: 10.1007/s00267-026-02558-2 (PMC13391702; doi:10.1007/s00267-026-02558-2)

# Title: Contesting and integrating multiple ways of knowing in collaborative natural resource governance

**Journal name**: Environmental Management

# Authors: Molly C. Levy, Vicken Hillis, Hailey Wilmer, Lauren M. Porensky, Dave Pellatz, Rebecca Som Castellano

*Corresponding author:* Molly C. Levy, Human-Environment Systems, Boise State University, 1910 University Drive, Boise, ID USA 83725

E-mail: mollylevy@u.boisestate.edu

# Supplementary materials–Appendices

## Appendix A. Summary of interview guide materials

The interview protocol was organized around several key thematic areas designed to elicit respondents’ experiments, perceptions, and practices related to collaborative natural resource management in the Thunder Basin ecoregion. The thematic areas informed the development of semi-structured interview questions and contributed to the framework for qualitative analysis.

**Table A1.** Thematic areas of inquiry used in interview guides

| **Interview Categories** | **Themes (Non-rancher)** | **Themes (Rancher)** |
| --- | --- | --- |
| Background/Experience | Relevant experience, connection to TBER, interactions with producers | Personal and ranch history, values, motivations, quality of life indicators |
| Goals | Goals, values, motivations | Goals, management priorities, rangeland and grazing decisions |
| Collaboration, conflict, and cross-group interactions | Sources of conflict in TBER, group identities, collaborative successes or challenges | Sources of conflict in TBER, group identities, collaborative success or challenges |
| Wildlife and conservation | Attitudes toward conservation | Impacts on ranching operations from wildlife, attitudes towards conservation |
| Knowledge and ways of knowing | Types of information accessed, sources of knowledge, knowledge gaps | Types of information accessed, information sharing, sources of knowledge |
| Variable weather and drought | Impacts of climate change and ecological disturbance in TBER | Impacts on ranching operations from climate change, ecological disturbance |
| Community | Types of organizations involved with | Types of organizations involved with, changes in community over time |

## Appendix B. Extended qualitative results–Full results narrative and supporting quotations

In this supplement, we provide the full, unabridged Results section from the study, including detailed qualitative analysis and extended participant quotations. This material expands upon the condensed findings presented in the main manuscript and illustrates quotes, nuanced examples, and subgroup variations that were omitted from the article for brevity. While the main text prioritizes synthesis and analytic framing, this appendix preserves the richness and complexity of participants' perspectives on different ways of knowing (WoKs), their interactions, and their influence on collaborative land management in the Thunder Basin ecoregion (TBER). Readers interested in the full range of stakeholder perceptions and contextual detail may find this extended version especially useful.

**Results**

This section first presents findings related to the types and sources of knowledge present in collaborative contexts, and how different WoKs inform different goals for management. It then examines how stakeholders perceive and interact with different WoKs, and how these interactions shape their perspectives on collaboration. Excerpts from the interviews are provided to illustrate how various WoKs are expressed and operationalized by participants.

##

## What types and sources of knowledge are present in the collaborative, and how do they inform different management goals?

### Types and sources of knowledge

Participants describe different WoKs that relate to their own training and background, reflecting broader tensions regarding land management in Thunder Basin. Although boundaries between these WoKs were not always distinct, three general categories of WoKs (scientific, local/experiential, and administrative) were consistently referenced and are described below (Figures 2-3).

*Scientific ways of knowing.* Scientific WoKs refer to knowledge developed through the scientific method, emphasizing empirical observation, reproducibility, and is verified through peer review processes. This WoK is typically acquired as a result of structured methods aimed at generating objective understandings of the world that can be generalized across different contexts.

Scientific WoKs were most frequently described by university-affiliated researchers and federal research scientists, who cited peer-reviewed literature as their primary source of information. These participants also engaged with professional networks and gray literature, blending formally-vetted data with professional judgement or opinion as a source of knowledge.

Although less directly involved in scientific research, participants from conservation NGOs and federal management agencies reported incorporating scientific findings into decision-making, especially in relation to ecosystem monitoring, regulatory enforcement, and program development. At the state and local levels, respondents were more likely to reference institutional networks and trusted colleagues as their source of information rather than engaging directly with the scientific literature.

*Local and/or experiential ways of knowing.* Local and/or experiential WoKs are based on firsthand, place-based knowledge developed through daily experience living and working in TBER. This WoK was most commonly referenced by ranchers and long-term residents, who described their understanding of the land as evolving through observation, trial-and-error, and knowledge passed down over generations.

Participants using this WoK emphasized the practical relevance of their observations, particularly in relation to forage conditions, weather patterns, wildlife presence, and rangeland health. Some also described collecting their own forms of longitudinal data, such as wildlife monitoring or informal mapping. These insights were often shared within family or community networks and supported through interactions with local extension agents or agency staff; knowledge verification occurs where networks collectively assess, refine, and reinforce knowledge based on shared practical outcomes and direct observation. While traditionally positioned in contrast to scientific WoKs, several respondents described emerging efforts to integrate local knowledge in collaborative decision-making. However, they also noted that this integration remained inconsistent and sometimes limited to informal interactions.

*Administrative ways of knowing.* Administrative WoKs refer to knowledge shaped by formal training, administrative structures, and policy frameworks governing natural resource management. This WoK includes understanding legal mandates, regulatory procedures, and bureaucratic processes that define permissible actions and shape agency decision-making. Administrative knowledge is validated through adherence to legal standards and policy compliance, as well as informal peer evaluation and job performance.

Respondents from federal and state agencies most frequently described this WoK, noting that their knowledge was shaped by academic background and professional experience. Many referenced using science-based assessments and technical reports, but emphasized that their decision-making was ultimately bounded by legal and administrative obligations.

Local agency staff also described administrative WoKs but emphasized their efforts to combine these frameworks with place-based knowledge and input from local residents. In addition, NGO staff and private consultants reported familiarities with agency processes and used this knowledge to support regulatory compliance, inform landowner outreach, or advocate for particular management outcomes.

### Ways of knowing inform varied management goals

Stakeholders across groups described a general commitment to achieving balance in land and resource management. However, their interpretation of balance varied, reflecting differences in how goals were informed by different WoKs and shaped by institutional roles, land tenure, or organizational mandates.

*Local knowledge and management goals*. Producers often described management goals that emphasized a desire to balance economic livelihoods with long-term land stewardship. Their goals corresponded to their localized, site-specific knowledge of the owned and leased lands used in their operations, and included attention to soil health, water availability, wildlife, and overall rangeland functionality. One participant summarized this perspective by stating,

“My goal is just to make it as functional and make it better… anything I can do to help the range, help the water, the wildlife, all of it… just trying to improve what's here, and sustain a living.” - TB02 (producer)

Their goals were also influenced by their deep commitment to sustaining the land for future generations. This was exemplified by one participant, who stated that

“I mean we’ll probably keep ranching anyway… Well you know, there, that story, you know, they say what do you do with a million dollars, well, I would just ranch until I was broke.”- TB03 (producer)

While many producers valued conservation outcomes, others expressed concern about management approaches that they felt did not align with their operational realities. Prairie dogs, for instance, were frequently described as “thieves” that diminish forage availability, reflecting tensions between species protection and working lands viability.

*Scientific knowledge and management goals.* Respondents working in scientific roles often described management goals informed by research priorities focused on broader ecological processes and regional-scale conservation outcomes. Many operated from a public lands framework, emphasizing the need to balance multiple uses while managing for ecosystem functions such as biodiversity, wildlife habitat, and long-term landscape resilience. Rather than aiming to optimize agricultural production, scientists emphasized identifying strategies that could meet both public conservational goals and land users’ operational needs. As one federal scientist explained:

“I come at rangeland management from the perspective of public lands, in that they have multiple objectives. So these are lands that are not just managed for livestock production or beef production. They're managed for multiple ecosystem services and so most of my research has been trying to address trade-offs or synergies in … achieving those ecosystem services.

So I'm not working with ranchers trying to help them increase their beef production. I would say I'm more working with them... with the ones who are more associated with public lands and having to deal with regulations related to wildlife and trying to work with them in terms of, well, how do we achieve both outcomes, both what they want and what the public wants for wildlife.” -TB10 (federal research)

This perspective positioned scientific knowledge as a tool for navigating complex, multi-objective systems rather than advancing a single land use goal. Scientists commonly sought to inform management strategies that support both ecological sustainability and working landscapes within the regulatory and ecological constraints of public lands.

Several participants also described efforts to connect scientific research with local observations and observed that integrating scientific and local WoKs enabled them to identify locally-relevant questions and consider alternative interpretations of their data. Participants noted that engagement with experiential knowledge had the potential to expand the scope and relevance of their scientific inquiries. As one researcher explained:

“It's a much different perspective when you try to work collaboratively. And you have to, again, take that ego out of the way and say, ‘You know, I think maybe I misinterpreted that data, or I didn't see your viewpoint or, boy, because of what you asked questions about, we now have a much more robust explanation of what went on…’

Having those scientists be able to understand the experiential knowledge and visit and hear what the pressing questions are, I think really opens up the door to take new skill sets, to take new disciplines, and apply that scientific power to help answer questions that might have been unresolved before, or maybe never even been asked because we didn't have that sort of scientific expertise.” - TB09 (federal scientist)

*Management goals associated with agency WoKs.* Participants from federal, state, and local agencies described goals informed by both scientific information and administrative or legal mandates. When asked about their sources of information, participants described information-sharing that occurred across agencies through technical reports and informal communications, as well as interactions with community members and other stakeholders:

“I go to workshops, and I talk to anybody with expertise. I talk to Game & Fish and Fish & Wildlife. I talk to the Prairie Dog Coalition, and I, you know, I, I... I don't just read off the internet. I find people with expertise in the area.” -TB40 (county agency)

Federal agents also described a goal of “walking that balance” in regards to simultaneously maintaining healthy wildlife populations while also managing for multiple land uses. This balancing act was framed as navigating a “sweet spot” of public acceptance, wherein both producers and conservationists would need to compromise: livestock producers might tolerate more prairie dogs than preferred, while conservation advocates would accept areas where prairie dog control remains necessary. Respondents also underscored their goal of cultivating respectful relationships with landowners, often expressing sincere concern for how federal actions affect their partners and communities. Despite operating under federal mandates, many reside in the surrounding community and emphasize their commitment to building trust and understanding at the local level. One participant described the tensions between scientific recommendations and the lived realities of landowners, stating:

“So, the science says this and the science says that. Then when we're out there dealing with landowners and they say, ‘But I can't make a living if we're doing it this way.’ Those are kind of really big challenges, when you're dealing with someone's livelihood, you can't always just consider the science. There has to be that balance between that cultural need and that scientific need in there, and I oftentimes feel like… we’re the ones that end up dealing with the brunt and both sides of it… You can't always go one way or the other, because if you're talking about someone's livelihood, we can't say, ‘Well we're going to manage it this way because scientifically, ecologically, that makes the most sense, but we're going to put you out of business. Sorry.’” - TB16 (federal management)

##

## How do stakeholders perceive and engage with different ways of knowing in the context of collaboration?

### Collaborative workshops and working group meetings provided opportunities for participants to engage with perspectives informed by different WoKs. While collaborative processes were often described as mechanisms for integrating diverse knowledge systems, several respondents identified persistent tensions concerning the credibility and legitimacy of different types of knowledge. The following sections examine how participants perceived and engaged with different WoKs within these collaborative contexts.

### Perceptions of scientific WoKs

*Scientific perspectives.* Participants with scientific training or institutional affiliations in research and conservation emphasized the importance of decision-making grounded in peer-reviewed scientific data. These respondents frequently referenced the role of formal methods in minimizing observer bias and producing replicable results. One researcher explained:

“But there's a scientific process for a reason because we all have observer bias. And that scientific process, as you know, is there to try to extract that, separate that observer bias from the objective reality, and so I think that if I was just to say what I see, am I right? I don't know.” -TB18 (university research)

Several participants acknowledged the value of local WoKs, but expressed frustration when other working group members either questioned the validity of scientific information, or didn’t grant it authority over other WoKs. These respondents acknowledged the importance of ranchers’ experiential knowledge and observations of the land, but ultimately emphasized that scientific data should take precedence in shaping management recommendations:

“There's not a lot of science… The ranchers have different ideas on the science because they live there. So, it's like you want to engage them and their understanding of the land and what they're observing, because those observations are important, but at the same time we have real data from those scientists like yourself to contribute. And when those discussions, when that, when that doesn't get properly introduced or considered for a recommendation, it's really frustrating.” -TB12 (conservation)

*Management perspectives*. Although participants involved in land management discussed the value of scientific knowledge, many agency personnel, particularly those managing at the local level, did not frequently reference scientific literature as a source of information unless specifically prompted. Many participants instead described indirect engagement with the literature by relying on colleagues or institutional networks to interpret and apply scientific findings. Conversations often reflected the perception that scientific interpretation was outside of their core responsibilities. One participant described their reluctance to overstep their professional boundaries, stating:

“It's important to me from a perspective of, I want everything to be based on good, sound science. That being part of my background, I try to be a little in tune to it. It's a little bit out of my purview because again, I've got a colleague at Game & Fish that's much more of an expert than I am on that, so I'm relying on that. Or if it's some rangeland studies about grassland productivity and those interactions with prairie dogs, I'm talking to my guy in the Department of Ag, ‘Hey, have you heard about this? Or what's going on here? Is that a good paper?’ Connected, but also I'm in my lane as well.” -TB25 (state management)

*Producer perspectives*. Agricultural producers in TBER described engaging with scientific research primarily through their participation in ecological studies or collaborative research projects, although the degree to which they were willing to engage varied with the perceived utility of the research (Wilmer et al., 2025). Several participants expressed uncertainty about how research findings translated into practical outcomes. As one producer reflected:

*“*In the long run, I am not sure how much of that information gets put to practical use. Which is the same way with a lot of research and studies and surveys is that, that data is all there, but unless it has… some way of getting up from here to the people that actually are making the difference, um, that’s the hard part, that bridge from there to there is the hard part to do.” - TB03 (producer)

Other respondents noted that participation in research was more about increasing the legitimacy of their own local knowledge by validating it through scientific studies, rather than filling in an information gap. One producer remarked:

“Data is nice, but sometimes data is not prudent to what we do. That’s just kind of like this drought study they are doing up there–yeah we know that drought is detrimental and I know that without the data. Even in the drought study, I know from practical experience because I’ve been here for years and years that there is a timeframe if we don’t have moisture that, that we’re in trouble … I don’t have to have a scientific study to tell me that … but most of the ranchers already know that. So as far as that study really helping and benefiting a rancher, probably not. I mean it makes nice data, but in the end we know that already. We don’t have to have it scientifically proved because we’ve proven it our self.” - TB43 (producer)

And another producer noted:

“You know they have things like, they call today ‘holistic resource management’, HRM. And they have HRM schools. And I’ve looked at those schools and studied what they wrote and everything and their teachings, and it makes sense. What I found interesting, again at the risk of sounding arrogant, is [my great-grandfather] wrote that book, before it came out in the 70’s or 80’s.” -TB04 (producer)

### Perceptions of local WoKs

*Producer perspectives.* Ranchers often prioritized their own local knowledge, which is rooted in multi-generational histories, lived experiences, and firsthand observation of ranch, landscape or regional scale dynamics. These respondents referenced their lived experience as central to understanding context-dependent ecological conditions and guiding management decisions that shape the lives of plants, animals and people. One participant summarized this view:

“They know how it’s going to work best for their property and how it’s going to work best for their animals because everybody’s animals and property [are] different. And if they’re generational and if they’ve been there quite a long time, then they're going to have all of that technical knowledge already.” -TB03 (producer)

The speaker emphasizes the site-specific variability that makes it difficult for generalized scientific recommendations to fully capture the nuances of what works best on individual operations. The statement that producers “already have all of that technical knowledge” suggests that, especially for generational ranchers, this local knowledge is not only as valid as formal scientific knowledge, but also more relevant when it comes to day-to-day decision-making.

*Agency perceptions of local knowledge.* Agency personnel, who are responsible for land, wildlife or other resource management decisions at a large scale, acknowledged the value of local knowledge in understanding on-the-ground conditions and place-based management practices. Some described relying on producers for updates on forage conditions, wildlife presence, and land use practices. One federal employee recalled:

“I always have listened to the ranchers and actually relied on them for a lot of information on what's happening on the ground. I think the Forest Service is getting better at that. At the time, going through this, I had a district ranger that believed in it very much… He came in, and he goes, ‘You guys are really arrogant.’ What do you mean? He goes, ‘You think the only good data is Forest Service data or what you know.’ He said, ‘You got to start listening to the people that live out there.’” TB24 (federal management)

This perspective was echoed by others who described the limitations of relying solely on agency-generated or peer-reviewed data, recognizing the need to incorporate more localized forms of expertise and to sustain long-term relationships with private-sector land management partners. Conversely, some agency respondents reflected on the challenges of meaningfully incorporating locally-collected information into formal processes, especially when it lacked the structure or perceived rigor of scientific studies. One county official stated:

“We definitely have some producers that over the years have collected their own version of information. Again, it's hard. So if you're going to say, well, the standard for what we're going to accept is something like a peer reviewed study, none of their information will ever qualify. But is it worth throwing completely out? That's hard. They're trying to communicate their information with you, and it's sometimes not as tangible as I would like it to be.” - TB32 (county agency)

These considerations demonstrate the broader disconnect between recognizing the value of local WoKs and having the means to incorporate it into formal decision-making. Still, some agency staff advocated for pathways to better support the communication and translation of producer knowledge.

*Scientists’ perceptions of local knowledge.* Scientific researchers recognized the value of local WoKs, but emphasized distinctions between anecdotal observation and scientific expertise. One researcher described concerns over how local knowledge was being framed at a public meeting:

“When some of the ranchers at the meeting were making comments, they were saying, ‘Well my research shows the following.’ And what is their research? Is it published research? What do they mean by that? I feel like people were presenting themselves as having scientific expertise and knowledge, that really absolutely are not scientists [or] trained as scientists, so it's like saying you're a heart surgeon when you're not… You could ask some of these people what their source is, and they might say ‘Well I've done my own research, and I've done this’, and what does that actually mean? I don't know. But in my case, mine's peer-reviewed scientific research.” -TB18 (university)

This respondent continued by describing how both scientists and ranchers are susceptible to observer bias, and emphasized the role of scientific methods in producing verifiable knowledge:

“If a rancher says what they see, are they right? I don't know. I mean, we can't really know unless you're actually going to do some research. I do think there's Indigenous knowledge, there's knowledge that people have whether they're an ecologist or a rancher because they've been spending time in that system. Is that valuable? Yes. Of course. Does it mean it's all correct? No.” - TB18 (university research)

Across interviews, participants illustrated a tension between scientific perspectives and other WoKs: while local knowledge is acknowledged as meaningful, its credibility is frequently questioned when it is not generated through formal scientific processes.

### **Interactions between different ways of knowing**

*Clashes between ways of knowing*. Participants described a range of interactions between scientific and experiential WoKs, with several interviewees referencing tensions that emerged around specific scientific findings. In one example, participants referenced a study that found prairie dog colonies may enhance forage quality under certain conditions (Connell et al., 2019b). This finding was interpreted in varying ways in relation to the perspectives, knowledge, and experiences of those engaging with it.

For some conservation-oriented respondents, the study reinforced their existing understandings of prairie dog ecology, specifically that the moderate levels of disturbance produced by prairie dog activity may enhance forage quality and benefit grazing livestock in certain conditions. For these respondents, the science aligned with their ecological values and long-term conservation goals. As one participant explained:

“At certain levels, prairie dog colonies will actually increase the nutritional value of the vegetation that is on their colonies, through many processes. There may be less available forage for cattle, but every bite they take is more efficient, and more nutritious… So for example, if 10 percent of an allotment were occupied by prairie dogs, there could be a net benefit [to grazing livestock].” -TB11 (conservation)

By contrast, several landowners expressed skepticism toward the findings, because the conclusions didn’t align with their lived experiences. One producer rejected the research as “hogwash,” not because the ecological mechanisms were misunderstood, but because the findings felt disconnected from the visible, material conditions they were navigating. As the producer further explained:

“She had a big research paper and said, ‘There's more production on prairie dog towns because they chop it off and so it's higher protein and all this.’... I mean, what she's saying may be true but it's all that tall [makes hand gesture denoting very short].” - TB21M2 (producer)

Other participants viewed the study with more nuance, reflecting on the potential disconnect between the reported findings and the reality of local conditions. As one federal researcher explained, the measurements were taken on small colonies at the edges of the complex, while many landowners were dealing with large, central colonies where ‘there was just absolutely no forage’ (TB10). The researcher went on to elaborate, saying:

“There was like this real disconnect between what people were seeing when they go out and visited these huge colonies, versus what [the research] was saying about these smaller colonies. So again, I think that did not go very well in our favor to seem like, what are these scientists doing? They're just saying things that don't even make sense.” -TB10 (federal research)

While not disputing the validity of the study’s methods or findings, the speaker acknowledged that the study’s credibility was compromised by the mismatch between the scale of scientific observation and the scale of stakeholder experience. From this perspective, the underlying issue wasn’t that the science was wrong, but that it wasn’t addressing the ‘right’ question regarding the conditions that stakeholders were most concerned about (i.e. visibly barren prairie colonies).

*Integrated ways of knowing.* In addition to points of divergence, participants also described instances where different WoKs were brought together in ways that supported mutual understanding and informed adaptive management. These examples were most commonly shared by individuals who had experience with collaborative and/or co-produced research, long-term engagement in the working group, or active involvement in extension or outreach activities. Several participants discussed how encountering experiential knowledge that differed from their scientific understanding prompted new lines of inquiry. One federal researcher explained:

“If a rancher tells me something that is surprising to me, then I want to go do a project on it… Whoever it is, if they're saying something like, ‘No, this is what I see out in the world, or this is what so and so told me,’ there's a reason for that, right? That's my approach–well, there must be something going on there. And just because I found something in my study doesn't mean, like what am I missing? … It makes me want to go bigger or go in a different direction usually, but it doesn't make me not believe my results. It just makes me think maybe I missed something else that's going on.” -TB13 (federal research)

This perspective was echoed by others who emphasized the importance of acknowledging instances when scientific results do not match stakeholder experiences, and highlighted the importance of local context and long-term observation in interpreting scientific findings. Similarly, another federal land manager emphasized the importance of reconciling divergent observations by actively asking what might be missing between scientific and local observation:

“In my mind I always think, ‘Okay, what's missing? Because science is saying this, but their local knowledge, and their history is saying this.’ And those don't always line up… You can't just come and do research for two or three years and answer a question that may have taken 50 years, 70 years of watching the landscape change and get an answer, and so sometimes that cultural aspect, that history within families is so vitally important, because we may not be able to answer it scientifically. I think it's really just trying to marry those things together because it's on both sides. Both of those sources of information are important.” - TB16 (federal management)

Some participants described how collaboration had shifted their perspectives on research, citing the advantages of being able to work directly with scientific researchers to address what information would be useful for management. One agency representative noted:

“I will admit, I was not a fan of research, having not had researchers ever really ask what management needed. And then they come in with this data, and it's like… that's not helpful. Then they would walk away with the data too, a lot of times.

That was one thing that was really neat about the grasslands is, for a long time now, we've been able to meet with the researchers and talk, and that's what they ask. ‘What do you need?’ They take our feedback… so now I'm a big fan.” - TB24 (federal management)

Respondents also acknowledged the role of diverse perspectives in shaping decisions and facilitating compromise. As one county manager put it:

“You need professionalism from all corners. And if you just sit back and listen to 'em, and if everybody will give a little, you can go a long way. I mean, you have your archaeologist; I could care less about it, but I can understand how important it is. I don’t understand why it takes so long to get a survey done, but that’s not my expertise, so I can sit here and not understand it. Your biologist, I think they go overboard, but they feel their job is just as important as mine. So I do think you need all of that. You need every bit of that from every corner.” -TB40 (county management)

Taken together, these accounts suggest that while tensions between knowledge systems were present, participants also identified conditions under which integration was possible. Across interviews, respondents described moments of shared inquiry, particularly when scientific findings and local observations diverged. In such cases, questions about what might be missing or overlooked served as a catalyst for continued investigation and dialogue. Participants associated successful integration with transparent research processes, openness to feedback, and sustained engagement across knowledge systems.

##

## How do these perspectives on knowledge shape participants’ understandings of success in collaborative governance? Participants’ reflections highlighted the central role of knowledge in shaping their experiences with collaboration. Rather than viewing knowledge as neutral, many described how it was selectively interpreted or strategically used by different stakeholders. These dynamics influenced both how participants engaged with the process and how they assessed its outcomes.

### Selective interpretation and strategic use of knowledge

Participants described instances in which both scientific and experiential WoKs were selectively referenced or emphasized to support specific land use or management goals. Rather than treating scientific or local knowledge as static or universally applicable, respondents frequently discussed how individuals framed evidence in ways that aligned with their own perspectives or desired outcomes.

The selective use of knowledge was particularly evident in conversations about controversial species such as prairie dogs, and their perceived impacts on wildlife and rangeland health. One state agency official described how individuals used different WoKs to support competing claims:

“Somebody comes in and says, ‘Well we know from studies that prairie dogs only do this.’ Then you have somebody else over here saying, ‘Well, but from my experience as the owner of those lands for 100 years, I could tell you that's not totally true.’” - TB25 (state agency)

In this context, both scientific and experiential claims are presented as authoritative, even when they led to divergent conclusions. The same respondent noted that the perceived value of a study often depended less on its methodology and more on whether it aligned with a stakeholder’s desired outcome:

“You have all those figures and of course in a situation with sides, and with desired outcomes, people are going to try and use those to fit their situation.” - TB25 (state agency)

This sentiment was shared by others, including a county agency representative who observed that stakeholder positions may also influence the framing of the research questions themselves:

“I think [the research] is reliable. But it's just like anything. If you are for expansion, that's where you're going to lean your research toward. If I was out there doing research on the damage, it would go the other way. So I think it all depends on what side of the line you're on.” -TB40 (county agency)

Some participants reflected on how these dynamics could influence collaborative discussions, particularly when knowledge was used to assert authority rather than invite dialogue. One state agency respondent recounted a public meeting where conflicting observations about mountain plovers led to a contentious exchange:

“When people try and weaponize whatever knowledge they think they have, that's when you get into a bind… we had it happen when there was somebody who got up and said, ‘Oh I've seen plovers in tall grass. It's not like they don't use it,’ and somebody else got up and said, ‘You're full of crap. You don't know what you're talking about and I'm a scientist’ or whatever. That's where you do have a problem with that, as opposed to if the question is ‘I'm pretty sure I've seen a plover in tall grass. What do you know about that,’ and they go, ‘Don't know. I've never seen it. We've never studied it though so maybe it's possible.’ That's much different because you're bringing two pieces of knowledge together there to try and actually have that conversation rather than ‘I know this’ and ‘I know this.’” -TB27 (state agency)

This account illustrates that the manner in which knowledge is communicated can influence whether disagreements lead to conflict or collaboration. In this case, scientific knowledge became a tool of exclusion that was deployed to invalidate, rather than investigate alternative perspectives. In contrast, the hypothetical response posited by the speaker creates space for mutual learning by acknowledging the limits of both scientific and experiential WoKs.

In some cases, participants linked the selective use of knowledge to longer histories of tension around whose knowledge is granted legitimacy. A federal manager noted that repeated dismissal of local observations appeared to influence how landowners engage with ecological research:

“It doesn't matter what research says… and it may be just because they've gotten hammered on all these years. Every time [landowner] said, ‘We've seen them [wildlife] somewhere else," somebody said, ‘You're wrong.’” TB24 (federal management)

The participant suggested that, over time, such interactions contribute to a pattern in which landowners rely more heavily on personal or place-based knowledge, particularly when they feel their observations have been consistently challenged or discredited. As a result, some landowners viewed scientific findings as less relevant or trustworthy, especially when they do not align with firsthand experience, as described above. This pattern was described as a common point of friction in collaborative efforts involving multiple WoKs.

### Perceptions of WoKs influence attitudes toward collaboration

Interviews revealed an association between participants’ perceptions of different WoKs and their broader attitudes toward collaboration. Respondents who emphasized the value of multiple knowledge systems and expressed openness to learning across groups were more likely to view collaboration in a constructive light. These individuals often described collaborative spaces as opportunities to bridge gaps and co-create solutions to complex land management challenges. One conservation NGO representative described the potential opportunities that arose from working collaboratively across groups:

“How can we reach out to the people that have a lot to say in all of the silos of categories of people that we're working with to find out how to move forward now?... We have an opportunity, what are the steps to take? What are the barriers and how can we move forward? What are the needs? Forest Service still has to manage prairie dogs, landowners still live on the ground, we still want ferrets. Okay, so let's come up with a game plan and can we organize a group of people to create that game plan.” -TB20 (conservation NGO)

In particular, this account illustrates how assembling a group of stakeholders with diverse goals forced people to get out of their individual siloes, and instead consider how their individual expertise might advance the goals of the group. Other respondents reported similar observations, describing a shift among participants in how they approached collaborative discussions. One federal scientist stated:

“They’ve [producers] become engaged in a productive way that’s not just, how do we maximize production? It is, how do we sustain ecosystems and livelihoods in this region?” -TB13 (federal researcher)

This account was described in the context of ongoing dialogue within the working group, where participants were observed to broaden their framing of land management issues and consider how their decisions might affect diverse stakeholder interests.

Some participants acknowledged challenges associated with navigating bureaucratic processes, but still expressed overall satisfaction with the collaborative relationships. For example, one local government official noted that while the institutional pace and procedures could be a source of frustration, the collaborative effort was generally characterized by mutual respect and a willingness to work together:

“I think we get along great. And we're trying. My issue is, I am a local government, I'm a little guy. And so I'm used to if we need to do something, let's get it done now. And the federal government is not like that. They're slower, and that's where I can run into frustration. But as far as working together, I think we all work [well] together.” -TB40 (local government)

The participant went on to describe differences in institutional cultures and timelines, but also emphasized a practical approach to collaboration that highlighted adaptability and persistence in the face of procedural delays.

However, not all participants shared this optimism. Some individuals, particularly those representing scientific or conservation-focused perspectives, expressed dissatisfaction with the collaborative process when they perceived their contributions were not fully recognized. These participants often noted that when their knowledge was not granted sufficient legitimacy within group discussions, it led to feelings of exclusion and reduced confidence in the value of participating. One university-affiliated researcher described their experience in collaborative meetings as discouraging due to the perceived lack of appreciation for scientific input:

“From my perspective, I feel like these are our public lands, and that all of our voices matter. Scientists’ voices should matter regardless and so should like anybody from the public. I don't feel like that's being appreciated or respected or included, and so therefore... I was very disturbed by the meetings... I mean, these things are tough, but from what I experienced, from my limited experience, I would say I did not feel like that was a fair and public process.” - TB18 (university research)

Others shared similar frustrations and described how conversations around certain topics, particularly those related to wildlife conservation, were excluded from discussions. Respondents expressed concern that their perspectives were not given equal weight, and that this imbalance undermined the integrity of the process. The inability to fully pursue certain management goals led to feelings of discouragement and weariness among some participants. These individuals questioned whether the collaborative process was truly inclusive or capable of addressing the full range of stakeholder concerns:

“We're forbidden from discussing some of these realities… if we dare try to bring it up, we are told that's off the table… We're bullied basically into being quiet, and complying with the other side's desire to talk about how to get rid of the planned protections. Meanwhile, we have an environmental disaster occurring on the landscape, and nobody will even allow us to discuss it… I mean, this is not a consensus, or a real effort to try to find common ground. It simply isn't. Our basic needs are being refused to even be discussed.” -TB11 (conservation NGO)

Perceptions of whose knowledge counts and how it is expressed also played a role, as some respondents suggested that the collaborative process was often dominated by the most vocal stakeholders. These louder voices tended to crowd out more moderate or conciliatory perspectives, and subsequently individuals who were more open to integrating different knowledge systems were perceived as less visible in shaping group decisions. One federal agency representative reflected on this imbalance, noting that those willing to compromise often remained unheard:

“The people that are the ones that are really willing to compromise aren't the loudest… Oh, you know, the old expression, ‘The squeaky wheel is the one that gets greased’? And that's kind of how it feels… the ones that are making the most noise are the ones that get addressed, and yet we have a huge, like 98% of the folks that we deal with are these outlying groups, and they really aren't so polarized. They're much more, where they can kind of come to the middle and work together. But, we have a few distinct folks on each side that make it very, very, very polarized.” - TB16 (federal management)

Such accounts illustrate how participants' perceptions of whose knowledge was valued shaped both their attitudes toward collaboration and their sense of whether it could lead to meaningful outcomes. While some remained hopeful about the potential for inclusive decision-making and working toward shared solutions, others reported an adversarial dynamic when they felt particular WoKs were dismissed or sidelined. These varied experiences reflect the complexity of collaborative efforts in multi-stakeholder contexts and provide the basis for further interpretation in the discussion that follows.

## Appendix C. Stakeholder archetypes by epistemic openness and goal commitment in collaborative governance

**Fig C1.** Conceptual diagram illustrating how stakeholder *openness to diverse ways of knowing* (epistemic openness) and *strength of commitment to one’s own goals* influence collaborative governance. Four archetypes emerge: **Dogmatic adherence** to a single epistemology; **Pragmatic advocacy** leveraging multiple knowledge forms; **Procedural technician** constrained by institutional mandates; and **Curiosity-driven collaborator**.

Colored shading indicates the anticipated impacts on collaborative effectiveness–**Red:** likely negative, can lead to persistent conflicts; **Yellow:** Minimal to negative, may not cause conflict but limits meaningful collaboration; **Green:** likely positive, promotes trust and social learning; **Gradient:** Mixed/contextual, effective if knowledge integration is genuine but may risk mistrust if selective evidence is perceived as manipulative.


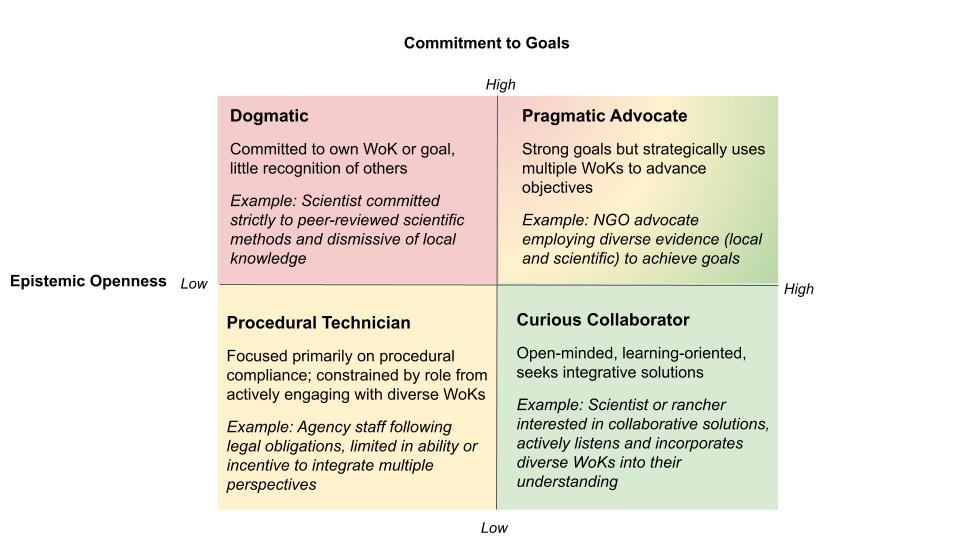

Supplement: Supplementary file 1 — Levy_SupplementaryMaterial_EM [file 267_2026_2558_MOESM1_ESM.docx]
